# Dysferlinopathy as cause of long-term hyperCKemia with preserved strength

**DOI:** 10.1186/s13023-025-03850-w

**Published:** 2025-06-22

**Authors:** Ikreet Cheema, Jacob Goodwin, Teerin Liewluck, Robert Charles Bucelli, Alan Pestronk, Margherita Milone

**Affiliations:** 1 Department of Neurology, Mayo Clinic, Rochester, MN 55905 USA; 2 Department of Neurology, Washington University, St. Louis, MO USA

## Abstract

**Background:**

Dysferlin (DYSF) has a crucial role in sarcolemmal repair. While *DYSF* mutations commonly manifest as limb-girdle muscular dystrophy (LGMDR2) or distal Miyoshi myopathy, atypical manifestations, such as asymptomatic hyperCKemia and pseudometabolic myopathy, are rarely reported. We describe clinical, serologic, radiologic, genetic, and muscle pathology findings of three patients with rare dysferlinopathy phenotypes and long-term follow up in one of them. We also review the literature pertinent to uncommon forms of dysferlinopathy presenting with hyperCKemia and pseudometabolic phenotype.

**Results:**

Patient 1 is a 51-year-old female with exercise-induced myalgia predominantly affecting calf muscles for 7 years. She had a 22-year history of asymptomatic hyperCKemia (CK 812–2,223 U/L). Neurologic exam showed mild calf enlargement without weakness. CT of the lower limb revealed fatty infiltration of distal peroneal and calf muscles. Genetic testing showed two *DYSF* variants, c.2163-2A > G (pathogenic) and c.866C > G, p.Ser289Cys (VUS), unknown if heteroallelic. Muscle biopsy demonstrated nuclei internalization and absent dysferlin immunoreactivity. Patient 2 is a 20-year-old male, football player, with an episode of exercise-induced myalgia followed by asymptomatic persistent hyperCKemia (729–2,645 U/L). He had normal strength but mild calf muscle atrophy. Muscle MRI demonstrated subtle T2 hyperintensity in the posterior leg compartment musculature. He has two heteroallelic *DYSF* variants, c.6008G > A, p.Gly2003Asp (pathogenic) and c.854C > T, p.Thr285Met (VUS). Muscle biopsy showed no myopathic changes but reduced dysferlin immunoreactivity. Patient 3 is a 58-year-old female with incidentally detected asymptomatic hyperCKemia (CK: 249–2,096 U/L) for 2 years. She had normal strength and normal lower limb muscle MRI. She carries two heteroallelic *DYSF* variants, c.2517del, p.Met840Trpfs*108 (pathogenic) and c.6058C > T, p.Arg2020Cys (VUS). Muscle biopsy showed minimal myopathic changes and attenuated dysferlin immunoreactivity. Reduced dysferlin expression was confirmed by western blot in patients 2 and 3. Needle EMG was normal in all patients.

**Conclusions:**

Dysferlinopathy should be considered in the differential diagnosis of metabolic myopathies and asymptomatic hyperCKemia. Patient 1’s long history of hyperCKemia without weakness over two decades suggests that CK elevation in dysferlinopathy does not necessarily predict development of weakness. Additionally, the lack of dystrophic changes on muscle biopsy of patients with asymptomatic or minimally symptomatic hyperCKemia should not discourage the search for dysferlin deficiency in muscle, particularly in the setting of *DYSF* variants.

## Introduction

Dysferlin (DYSF) is a sarcolemmal protein with a crucial role in calcium-dependent sarcolemmal resealing and repair [1]. Mutations in *DYSF* lead to protein loss or dysfunction resulting in a defective sarcolemmal repair process and consequent muscle degeneration. Dysferlinopathies, which are autosomal recessively inherited, are phenotypically diverse but most commonly manifest with limb-girdle muscular dystrophy (LGMDR2) or distal myopathy which predominantly affects posterior leg compartment muscles (Miyoshi myopathy) or, less frequently, tibialis anterior. Asymptomatic elevated creatine kinase (hyperCKemia) and a “pseudometabolic” phenotype (not just as symptom onset), characterized by exercise intolerance, myalgias, and rhabdomyolysis, are exceedingly rare dysferlinopathy presentations with very few patients reported in the literature.

Here we describe the clinical and laboratory features of three patients with dysferlinopathy manifesting with a rare phenotype to increase awareness about these rare disease manifestations and accelerate diagnosis. We also review previously reported dysferlinopathy patients with unusual presentations of hyperCKemia and pseudometabolic phenotype.

## Methods

We analyzed the clinical, serological, electrophysiological, radiological, genetic, muscle biopsy histochemical, immunohistochemical and western blot findings of three patients with rare dysferlinopathy phenotypes. We also reviewed the literature for previously published dysferlinopathy patients with hyperCKemia and pseudometabolic phenotype and summarized the findings. The study was approved by the Mayo Clinic Institutional Review Board.

## Results

### Patient 1

**Fig. 1:**
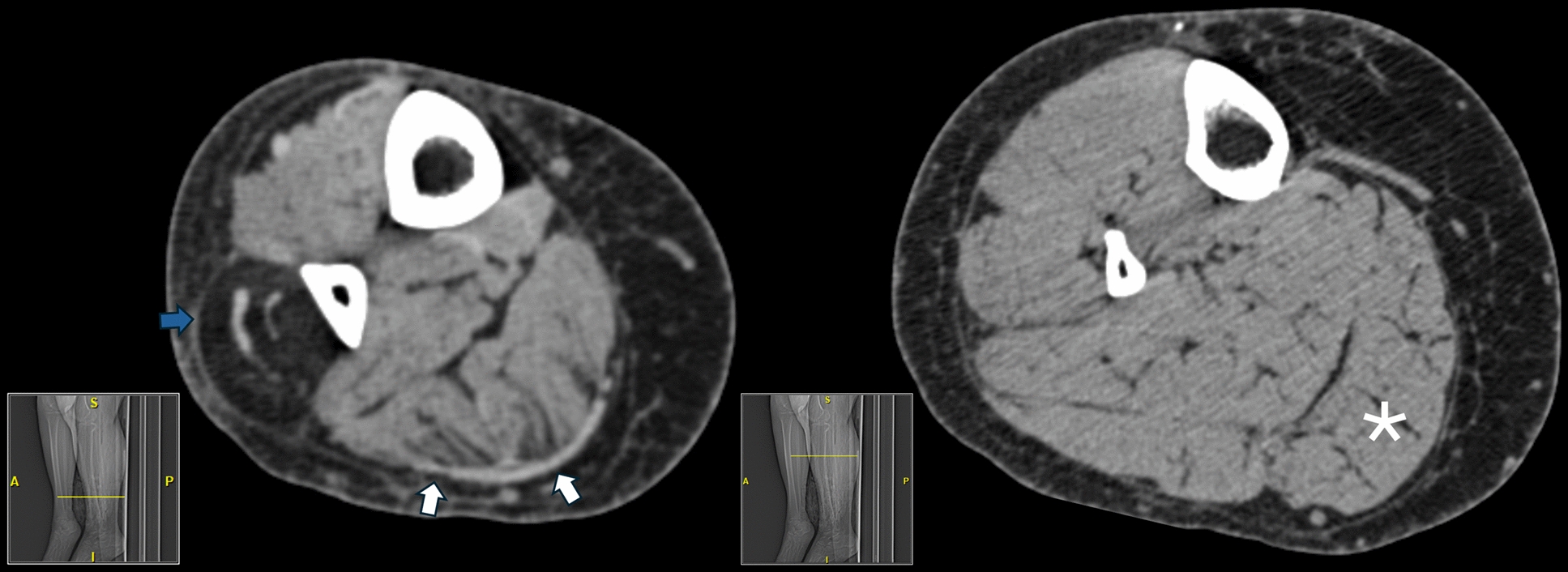
Patient 1's CT images. Non-contrast axial CT of the right lower leg (left) revealing hypodense fatty infiltration most prominently of the distal peroneal musculature (blue arrow) with mild muscular marbling of the soleus and gastrocnemius (white arrows), which is spared more proximally (right, asterisk). A = anterior; S = superior; P = posterior; I = inferior

**Fig. 2:**
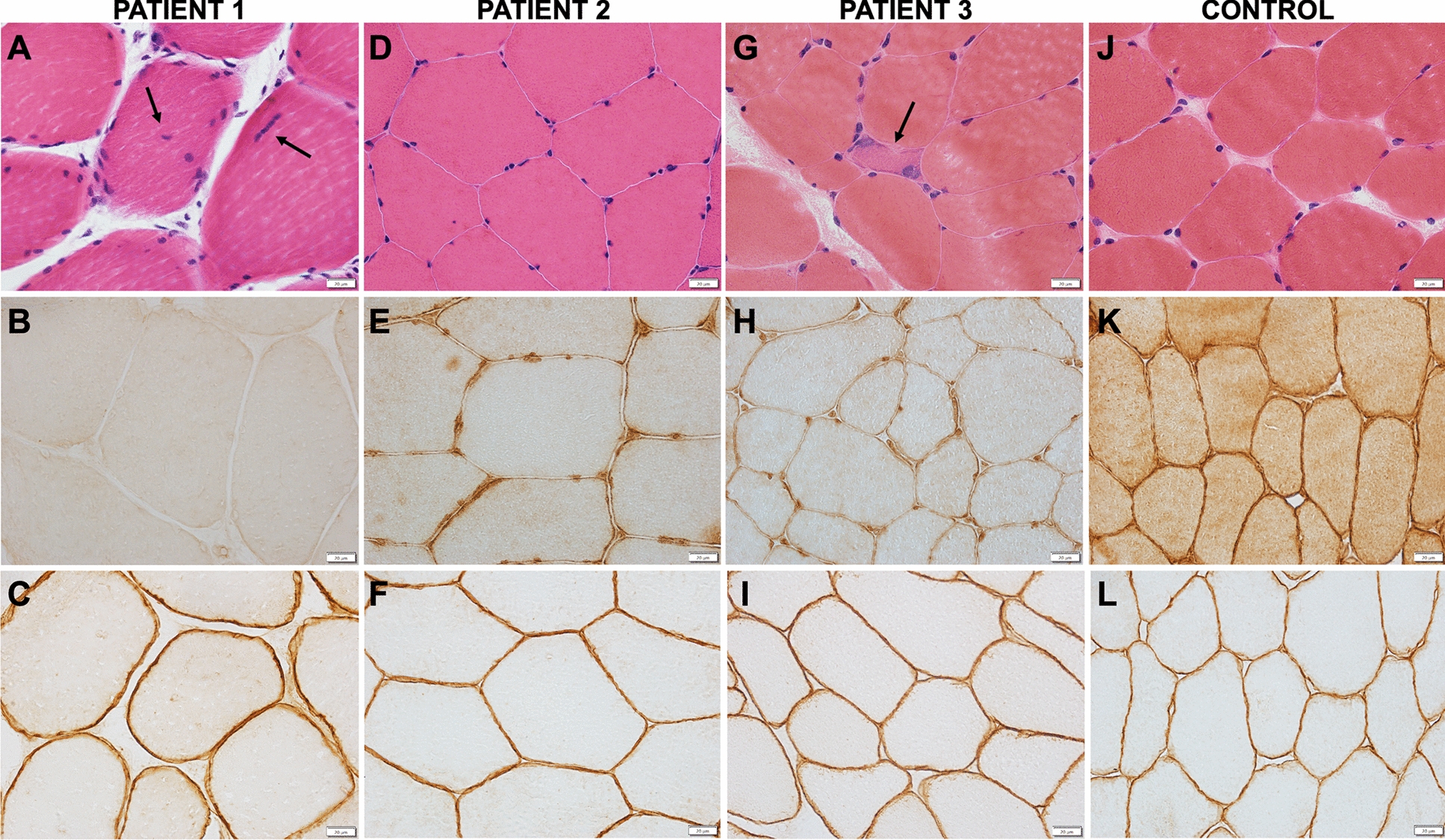
Muscle biopsies. Patient 1 (A-C): **A** Hematoxylin–eosin, **H** and **E** stained section showing internalized nuclei (arrows, nuclei staining blue); **B** sarcolemmal dysferlin immunoreactivity is absent compared with normal control **K**; and **C** normal sarcolemmal caveolin-3 immunoreactivity. Patient 2 (D-F): **D**, **H** and **E**-stained section showing normal findings; **E** sarcolemmal dysferlin immunoreactivity is patchy compared to normal control **K** and **F** normal caveolin-3 immunoreactivity. Patient 3 (G-I): **G**, **H** and **E**-stained section showing a regenerating fiber (arrow); **H** sarcolemmal dysferlin immunoreactivity is reduced compared to normal control **K** and **I** normal caveolin-3 immunoreactivity

A 51-year-old female presented with exercise-induced myalgias predominantly affecting her calf muscles for 7 years. She was known to have asymptomatic hyperCKemia ranging from 812 to 2,223 U/L (upper limit 192 U/L) for at least 22 years prior to symptom onset. Clinical examination revealed normal muscle strength and mild calf enlargement. Plasma acylcarnitines, blood lactate and urine organic acids were normal. Antibodies associated with inflammatory myopathies and immune mediated necrotizing myopathy were negative. Needle electromyography (EMG) was normal in proximal and distal muscles. Next generation sequencing (NGS) targeting 230 genes causative of muscle diseases revealed two *DYSF* variants, c.2163-2A > G (pathogenic) and c.866C > G, p.Ser289Cys (variant of unknown significance, VUS). This latter variant is present in 0.004% of the population. Modeling output of the protein sequence and biophysical properties (performed by the commercial laboratory where testing was performed) did not meet statistical confidence threshold to predict the impact of this variant. It is unknown if the two detected *DYSF* variants are heteroallelic as the patient’s parents are deceased. The patient had a computerized tomography (CT) of the right lower limb within one year of presentation due to fibular fracture (performed the day after fracture) which revealed fatty infiltration of the distal peroneal muscles and milder signal abnormality in the posterior leg muscles (Fig. 1). Biopsy of the vastus lateralis showed marked internalization of nuclei without necrotic or regenerating fibers and absent sarcolemmal dysferlin immunoreactivity (Fig. 2A, B). Caveolin-3 immunoreactivity was normal (Fig. 2C).

### Patient 2

**Fig. 3:**
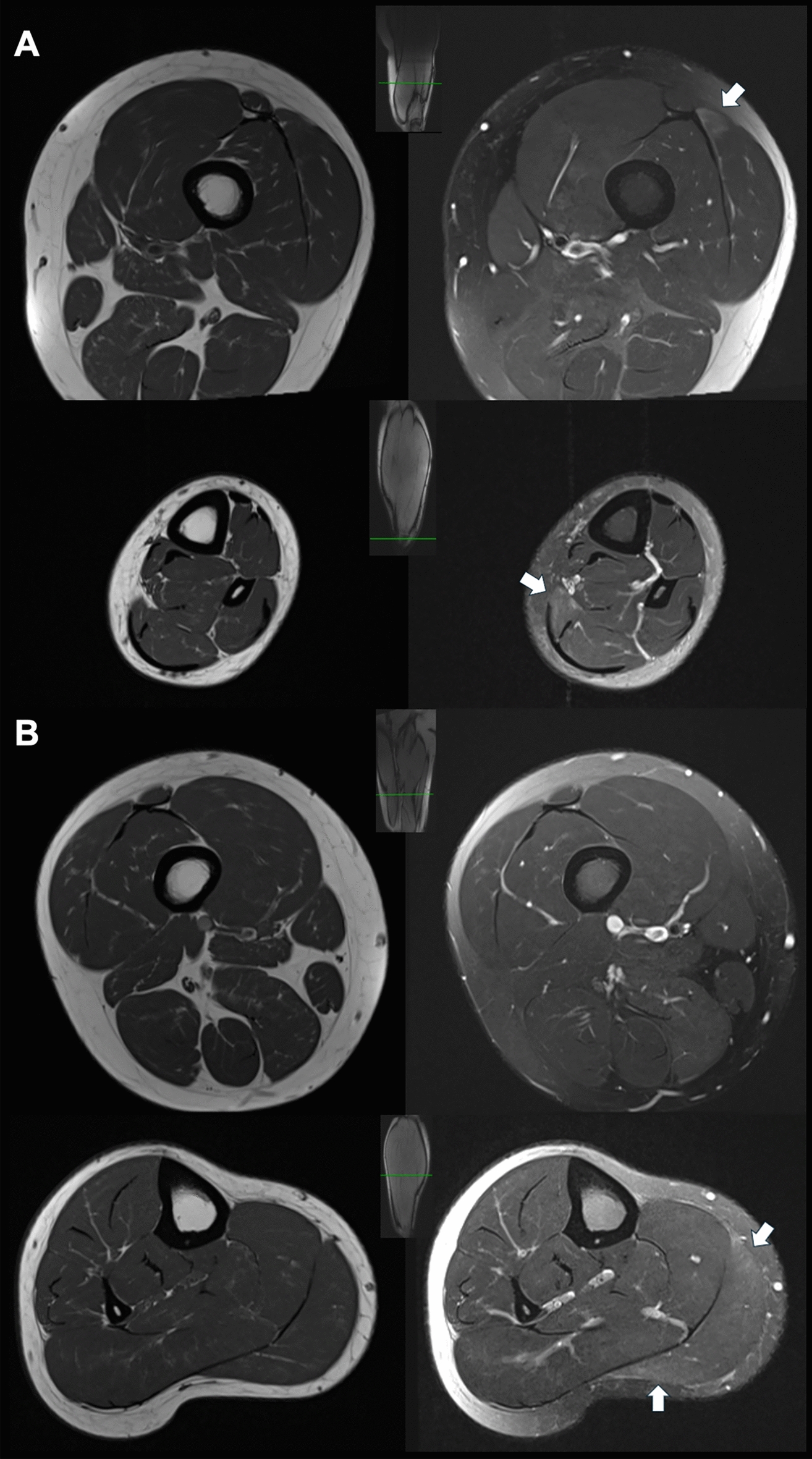
Patient 2's MRI images. **A** Left leg. Left images are T1 non-contrast axial MRI of the lower thigh (top) and lower leg (bottom) revealing no fatty infiltration. Right sided images are T2 STIR MRI of the right lower thigh (top) revealing subtle T2 hyperintensity in the vastus lateralis (arrow) and lower leg (bottom) revealing T2 hyperintensity in the soleus (arrow). **B** Right leg. Left images are T1 non-contrast axial MRI of the lower thigh (top) and of the lower leg (bottom) showing no significant fatty infiltration. Right sided images show non-contrast axial T2 STIR MRI of the right lower thigh (top) showing no significant abnormalities and lower leg (bottom) revealing T2 hyperintensity in the medial gastrocnemius (arrow)

**Fig. 4:**
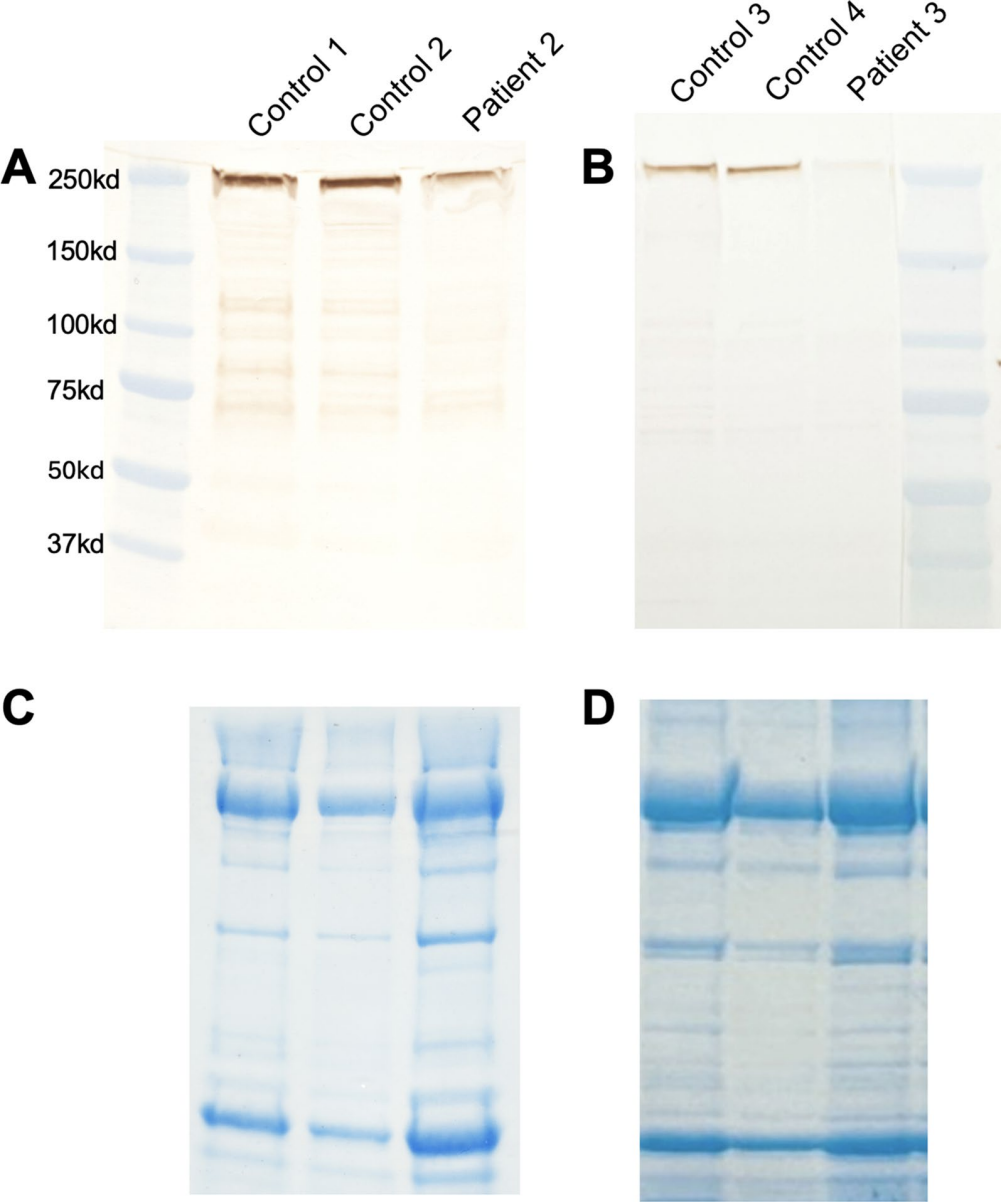
Western blot. Dysferlin western blots for patients 2 and 3. Panels A and B show the western blot utilizing a dysferlin antibody for patient 2 and patient 3, respectively. Both patients show reduced staining of the dysferlin band ~ 250 kd, as compared to the control samples. The lanes in Panels C and D for the Coomassie blue-stained gel demonstrate the total amount of muscle protein loaded on to the gel, corresponding to the above lanes in panels A and B, respectively. Panels C and D show that in fact there was more total muscle protein loaded on to the gel from both patients, relative to the controls, confirming a definite reduction in dysferlin amount

A 20-year-old male football player presented for evaluation of CK elevation, which was first detected after he developed lower limb muscle tightness and myalgias following a long football practice on a hot day. His CK was found to be elevated at 2600 U/L but did not normalize despite resolution of the symptoms. He subsequently had asymptomatic persistent hyperCKemia over 13 months of follow up (CK 729–2,645 U/L). On clinical examination, he had normal muscle strength, but mild atrophy of calf muscles compared to thigh muscles. Biomarkers of metabolic and immune-mediated myopathies were negative. EMG of the upper limb proximal and distal muscles, thoracic paraspinals, and lower limb muscles, which included tensor fasciae latae, vastus medialis, medial gastrocnemius, and tibialis anterior, was normal. MRI of the lower limb muscles demonstrated nonspecific findings suggestive of subtle edema in the posterior compartment muscles of both lower legs (Fig. 3). NGS targeting 230 genes causative of muscle diseases revealed two heteroallelic *DYSF* variants, c.6008G > A, p.Gly2003Asp (pathogenic) and c.854C > T, p.Thr285Met (VUS). This latter variant is reported in 0.007% of the population. Computational algorithms produced conflicting evidence regarding the predicted functional impact of this variant (REVEL score: 0.591) with PolyPhen-2 suggesting that this variant is likely to be disruptive. Whole genome sequencing (WGS) was further performed and did not reveal any pathogenic or potentially pathogenic variants in other genes causative of muscle diseases. Biopsy of the vastus lateralis showed no myopathic changes but a patchy reduction in sarcolemmal dysferlin immunoreactivity while caveolin-3 immunoreactivity was normal (Fig. 2D, F). Muscle dysferlin western blot confirmed reduced dysferlin amount (Fig. 4A) with normal calpain-3 expression (not shown).

### Patient 3

**Fig. 5:**
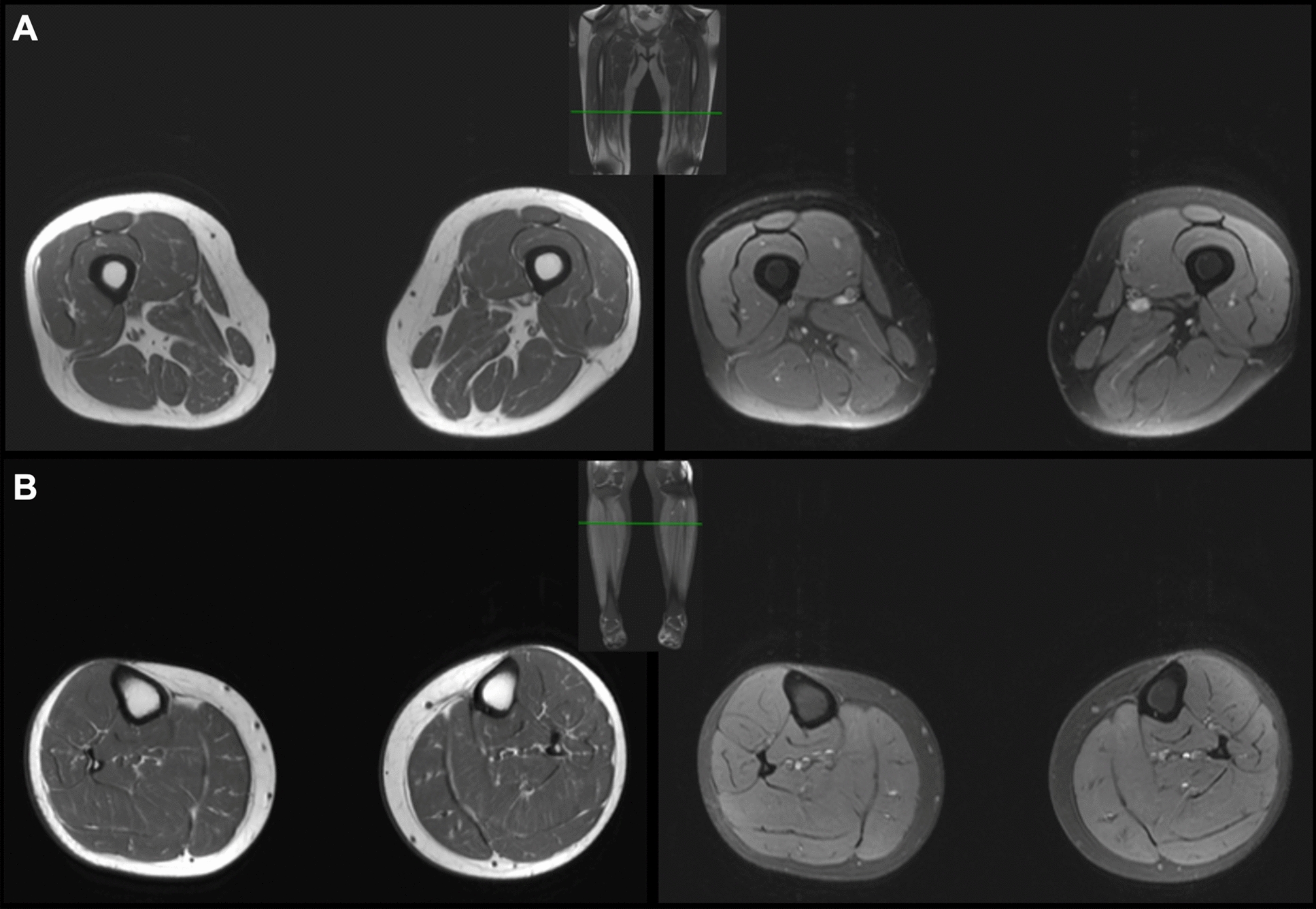
Patient 3's MRI images. **A** T1 non-contrast axial MRI of the bilateral lower thighs (left) and T2 STIR MRI (right) showing no muscle signal abnormalities. **B** T1 non-contrast axial MRI of the bilateral lower legs (left) and T2 STIR MRI (right) showing no muscle signal abnormalities

A 58-year-old female presented for evaluation of incidentally discovered asymptomatic hyperCKemia (CK 249–2,096 U/L; normal <170 U/L), persistent for at least 2 years. Clinical exam revealed normal muscle strength. EMG of first dorsal interosseus, triceps, deltoid, tensor fasciae latae, vastus medialis, gastrocnemius, and tibialis anterior was normal. MRI of the lower limb muscles showed normal signal intensity (Fig. 5). NGS targeting 230 genes causative of neuromuscular diseases revealed two heteroallelic *DYSF* variants, c.2517del, p.Met840Trpfs*108 (pathogenic) and c.6058C > T, p.Arg2020Cys (VUS). The VUS is present in 0.005% of the population and is predicted (PolyPhen-2) to be disruptive. Whole exome sequencing identified no additional pathogenic or potentially pathogenic variants in genes causative of myopathies. Biopsy of the vastus lateralis showed minimal nonspecific myopathic changes consisting of fiber size variability, one necrotic fiber, and rare regenerating fibers. There was patchy attenuation of sarcolemmal dysferlin immunoreactivity with preserved caveolin-3 expression (Fig. 2G, I). Western blot showed reduced muscle dysferlin (Fig. 4B).

## Review of the literature

Table 1 summarizes papers that have reported atypical presentations of dysferlinopathy, specifically asymptomatic hyperCKemia and the even more rare “pseudometabolic phenotype”. The described pseudometabolic phenotypes include distal leg painful swelling, myalgias, rhabdomyolysis, exercise-induced stiffness or intolerance [3–5, 8, 9, 11, 12, 16, 19–21]. The largest case series reported to date is a French cohort of 40 dysferlinopathy patients in which 10% of patients were diagnosed with pseudometabolic myopathy and 5% with asymptomatic hyperCKemia [3]. A few of these patients had only a single heterozygous *DYSF* mutation but the diagnosis of dysferlinopathy was supported by the lack of dysferlin in muscle. The overall reported prevalence of asymptomatic hyperCKemia in dysferlinopathy cohorts ranges from 1.6% to 15.4% [17, 18], while that of the pseudometabolic phenotype is approximately 8–10% [3, 11]. Sample sizes are, however, small limiting reliability of prevalence. The patient with the longest follow up in these cohorts was a 73-year-old female with exercise-induced stiffness and hyperCKemia over 12 years without muscle weakness [4]. Table 1 also includes patients with dysferlinopathy manifesting with hyperCKemia and transitioning to a more classic phenotype of LGMD or Miyoshi myopathy [2, 10, 13–15]. There is, however, limited data on the duration of hyperCKemia prior to development of weakness with intervals varying between 3 and 15 years [10, 13], and some patients were not followed up to mean age of dysferlinopathy onset [20].

**Table 1:** Summary of previously reported dysferlinopathy patients presenting with hyperCKemia or “pseudometabolic phenotype” and patients who progressed to typical forms of LGMD or Miyoshi myopathy

Authors (by year)	Report type	Diagnostic method	Phenotype	Description
Prelle et al. [2]	Case series	IHC and WB	HyperCK to MM	Italian cohort; 1/6 patients had asymptomatic hyperCKemia. 13-year-old female with CK 2,500 U/L, myopathic EMG, and absent dysferlin who developed Miyoshi myopathy 10 years later
Nguyen et al. [3]	Case series	IHC, WB, genetic testing (mutation list provided in paper)	PsMetab, HyperCK	French cohort; 4/40 (10%) pseudometabolic myopathy, defined as distal leg painful swelling without weakness or atrophy; 2 were misdiagnosed as polymyositis and 1 as focal myositis. 2/40 (5%) asymptomatic hyperCKemia. The latter were a 28-year-old female and a 50-year-old male, respectively, with CK elevation (15–30 × normal) with no muscle weakness or atrophy by age 58. All patients in both groups showed slight to severe posterior leg compartment abnormalities on MRI (unspecified type)
Klinge et al. [4]	Case report	IHC, western blot and genetic testing; c.1053 + 5 G > A and c.4411–5 C > G	PsMetab	73-year-old female with exercise-induced stiffness of the trunk and proximal leg muscles (no weakness) without progression over 12 years. At age 80, CK repeatedly elevated (1,906–2,412 U/L, normal < 150 U/L). Still ambulatory at age 85
Okahashi et al. [5]	Case report	IHC	PsMetab	18-year-old male with calf myalgias and rhabdomyolysis after exertion followed by persistent hyperCKemia (5,000–6,000 IU/L). EMG did not reveal myopathic changes. MRI demonstrated high short-time inversion recovery sequence (STIR) intensity in the gastrocnemius, soleus, semimembranosus, and biceps femoris muscles after symptom resolution
Fanin et al. [6]	Case series	WB and genetic testing	HyperCK	Muscle biopsies from 204 patients with asymptomatic hyperCKemia; 3 patients (2.2%) with CK > 1,000 U/L found to have dysferlinopathy
Paradas et al. [7]	Case series	IHC, WB, and genetic testing	HyperCK	1/29 patients (3.4%) had asymptomatic hyperCKemia; symptom onset at 27 years with no weakness after 5 years of follow up. On MRI, gastrocnemius medialis and adductor magnus showed increased STIR signal
Kobayashi et al. [8]	Case report	IHC and genetic testing; c.2997G > T and c.3373delG	PsMetab	28-year-old male with rhabdomyolysis after manual labor followed by asymptomatic hyperCKemia (around 2,000 U/L) for 10 years
Moody and Mancias [9]	Case report	IHC, western blot, and genetic testing; IVS17-1G > A, 2nd mutation not identified	PsMetab	15-year-old athletic male presenting with acute renal failure secondary to rhabdomyolysis followed by persistent hyperCKemia with 7 months of follow up (CK > 7000 U/L). EMG showed fibrillation potentials. MRI of the lower extremities revealed increased T2 signal intensity in the quadriceps, hamstrings, and gastrocnemius muscles
Li et al. [10]	Case report	IHC	HyperCK to LGMD	54-year-old female with a 3-year history of elevated transaminases. She had pitting edema and numbness in the lower limbs. She developed difficulty standing up and exercise intolerance. CK was 5,635 IU/L at highest. She was treated with steroids for polymyositis. Authors reported amyotrophy of the gluteus maximus but normal strength. EMG was myopathic. Muscle biopsy showed dystrophic changes and leukocyte infiltration. IHC showed severely reduced dysferlin. She was diagnosed with LGMD-2B
Xi et al. [11]	Case series	IHC, WB and genetic testing (mutation list provided in paper)	PsMetab, HyperCK	Chinese cohort; 3/36 (8.3%) patients with exercise intolerance, age range 12–25 years and symptom duration 1–6 years. 3/36 (8.3%) patients with asymptomatic hyperCKemia of which 2 males, age range 11–17 years, and symptom duration 4–10 years
Harris et al. [12]	Case series	WB and genetic testing	PsMetab, HyperCK	International cohort of 193 patients; 3% had clinical diagnosis of asymptomatic hyperCKemia (median 5 years duration) and 2% pseudometabolic dysferlinopathy; no individual details
Umakhanova et al. [13]	Case series	Genetic testing; c.TG573/574AT (p. Val67Asp), homoz	HyperCK to MM	Indian family follow up study. One patient was followed from age 6 to11 without developing clinical myopathy, despite elevated CK (580 U/L) and myopathic EMG. On re-examination at age 26 he had Miyoshi myopathy. It is unclear when weakness manifested
Contreras-Cubas et al. [14]	Case report	Genetic testing; c.3851C > T (p.Gln1160X) and c.5979dup in exon 53 (p.Glu1994ArgX3 fs)	HyperCK to LGMD	14-year-old male with incidental CK 26,372 IU/L. Quadriceps biopsy showed necrosis and endomysial/ perivascular lymphocytic infiltrates. EMG was myopathic. Patient was misdiagnosed with polymyositis. After treatment with immunotherapy, he developed proximal weakness and WES was performed
Folland et al. [15]	Case series	IHC and genetic testing; c.6207del (p.Tyr2070Metfs*4) heteroz.*	HyperCK to LGMD	Large family with AD hyperCKemia and late onset dysferlinopathy. Proband had hyperCKemia at age 33. Muscle MRI at age 42 showed fatty replacement of soleus, medial gastrocnemius, flexor digitorum longus, and flexor hallicus longus. Weakness onset at age 43 with inability to stand on toes. Three individuals developed muscle weakness and one myalgia. All those heterozygous for the variant had hyperCKemia
Katz et al. [16]	Case report	Genetic testing; c.2643 + 1G > A, heteroz.**	PsMetab	16-year-old female with recurrent rhabdomyolysis following vaccination/infection; no weakness and normal CK at baseline
Nashi et al. [17]	Case series	Genetic testing	HyperCK	Indian cohort of 124 patients and 1.6% had isolated asymptomatic hyperCKemia
Wang et al. [18]	Case series	IHC and genetic testing (mutation list provided in paper)	HyperCK	Chinese cohort; 4/26 (15.4%) with hyperCKemia; all males with age onset 22.0 ± 6.1 years (range 16–29 years) and disease duration 0–0.5 years. CK level 23.6 ± 25.4 × normal (range 6.0–61.3 × normal). 3 misdiagnosed with viral myocarditis and 1 with dermatomyositis
Belhassen et al. [19]	Case series	WB and genetic testing; c.3113G > A, homoz. and c.4200dupC, homoz	HyperCK, PsMetab	Tunisian cohort; 2/20 (10%) had hyperCKemia. First patient: male with onset age 39 (duration 5 years) and CK 920 U/L. Second patient: male with onset age 10y (duration 7 years) and CK 3,722 U/L. However, one had a slight deficit of tibialis posterior muscle (strength 4 +) with effort-induced calf pain
Bardakov et al. [20]	Case series	Genetic testing [7/8 c.TG573/574AT (p.Val67Asp), homoz.; 1/8 c.1852G > A; (p.Gly618Arg); c.6196G > A (p. Ala2066Thr)]	HyperCK, PsMetab	Dagestan cohort and one Russian case; 8 pediatric patients. One classified as asymptomatic and 7 oligosymptomatic. 5 patients had symptoms including calf muscle fatigue and 2 had post-exercise swelling and myalgias. One patient was described as having full strength while the rest were graded as 5- in varying muscle groups. All had elevated CK with asymptomatic patient CK being lower (249 vs 570 U/L). MRI showed fatty infiltration of medial gastrocnemius and soleus in all patients and of posterior thigh muscles in most patients. No follow up described
Sanchez-Casado et al. [21]	Case report	IHC, WB, and genetic testing; c.5 T > C (p.Leu2Pro) and c.4856dup (p.Cys1621Leufs*Ter29)	PsMetab	52-year-old female with 4 years of persistent generalized myalgia worse with activity; no weakness. CK 1,600–3,000. MRI lower limb showed fatty replacement on T1 in gastrocnemius, adductor magnus, vastus medialis, and semimembranosus

AD = Autosomal dominant; HyperCK = asymptomatic hyperCKemia; IHC = immunohistochemistry; LGMD = limb girdle muscular dystrophy; MM = Miyoshi myopathy; PsMetab = pseudometabolic; WB = Western Blot; WES = whole exome sequencing

* = Reported as dominant mutation

** = Phenotype was attributed to the heterozygous *DYSF* variant but patient had no muscle biopsy to confirm diagnosis

## Discussion

We present three adult patients with unusual manifestations of dysferlinopathy and long-term follow up in one of them. While most patients with dysferlinopathy initially present with weakness, our patients exhibited either exercise-induced calf myalgia or asymptomatic hyperCKemia, both rare presentations of this disease. Patient 1 has no weakness 22 years after the detection of asymptomatic hyperCKemia. To our knowledge, this is the longest follow up of a dysferlinopathy patient with isolated hyperCKemia without symptom progression, including preserved strength, reported in the literature. This patient demonstrates that asymptomatic hyperCKemia is not necessarily a prelude to the development of muscle weakness in dysferlinopathy and suggests that some patients with such a phenotype may remain free of weakness.

Patient 2 presented with acute onset of exercise-induced myalgia, but his CK values remained persistently elevated in the asymptomatic state and in the setting of normal strength and minimally abnormal calf muscle MRI. Bardakov and colleagues described two stages of pre-manifest classic dysferlinopathy: (1) asymptomatic, featured by elevated CK; (2) oligosymptomatic, characterized by elevated CK, signs of fatty muscle infiltration, exercise intolerance or slight objective muscle weakness [20]. There is, however, limited data on the duration of a premanifest state of dysferlinopathy, a feature that is likely influenced also by the genotype. Considering that patient 2 is young, that his follow up is only 13 months long, and that the mean age of dysferlinopathy onset is approximately 21.7 years [22], one cannot exclude that he will develop muscle weakness. He could be in the early disease stage of a classic dysferlinopathy phenotype. Patient 3 had incidentally detected asymptomatic hyperCKemia in mid-adulthood with a normal lower limb muscle MRI and has developed no weakness over 2-year follow-up. Although her follow up is relatively short, given that she is in her late 50s, with an age decades older than the mean age of dysferlinopathy onset, it is unlikely that she is in the pre-manifest stage of a classic dysferlinopathy.

Previously reported patients with atypical presentations of dysferlinopathy, including hyperCKemia and pseudometabolic phenotype, showed some degree of muscle MRI signal abnormality, most commonly in the posterior leg compartment [3, 5, 7, 9, 21]. A study that evaluated muscle MRI data from patients with genetically confirmed dysferlinopathy, including 5 patients with asymptomatic hyperCKemia, found that 181/182 patients had fatty replacement on T1-weighted images, with the gastrocnemius and soleus being the most commonly affected muscles, regardless of clinical phenotype [23]. MRI was proposed as an imaging biomarker for early disease detection in patients without weakness. Progression of muscle signal abnormality on MRI was indeed shown to correlate positively with disease duration and functional impairment [23]. Contrary to such observation, patient 1 had total replacement of the distal peroneal muscles and minimal fatty infiltration of the gastrocnemius muscles by radiological study, despite more than two decades of myalgia and hyperCKemia. The eventual symmetry of these radiological changes are unknown as only right leg imaging is available. EMG did not detect myopathic changes in any of the 3 patients, in keeping with the mildness of the phenotype.

Our findings reinforce the utility of assessing dysferlin expression in muscle by immunohistochemistry and Western blot confirmation to demonstrate dysferlin deficiency in patients with unexplained hyperCKemia, or myalgias and hyperCKemia, despite the lack of dystrophic changes on muscle biopsy. While genetic testing is the gold standard for the diagnosis of dysferlinopathy, the identification of VUSs, as seen in our patients, remains a diagnostic challenge. Additionally, the frequent lack of parental DNA in adult patients limits the ability to establish if the two genetic variants are in *trans*. In such cases, muscle biopsy can rescue the diagnosis. Although reduced dysferlin expression can occur in the setting of caveolin-3 deficiency, caveolin-3 expression was normal in all 3 patients by immunohistochemistry and no mutations were detected in the caveolin-3 gene (*CAV3*) or other genes causative of muscle disease in all 3 patients. Muscle biopsy with immunohistochemical studies and eventual immunohistochemical-driven western blot analysis can also be helpful in establishing the diagnosis in patients with autosomal recessive mutations impairing sarcolemmal protein expression other than dysferlin when segregation studies are not available (heteroallelic mutations). Dysferlin analysis in peripheral blood monocytes offers a less invasive diagnostic alternative, demonstrating high concordance with skeletal muscle findings by Western blot, and can be useful when muscle tissue is unavailable [24, 25]. This test, however, is not commercially available in several countries.

Some reports have outlined the misdiagnosis of patients with these rare presentations of dysferlinopathy. In one series, dysferlinopathy patients with “pseudometabolic” symptoms, manifesting with painful leg swelling, were misdiagnosed as having an idiopathic inflammatory myopathy or focal myositis [3]. In another series, patients with asymptomatic hyperCKemia were misdiagnosed with viral myocarditis or dermatomyositis [18]. Such misdiagnoses can be corroborated by the detection of an inflammatory reaction in muscle, a finding not unusual in dysferlinopathy [26]. These examples of misdiagnoses indicate an insufficient recognition of the mildest phenotypes of dysferlinopathy which, in turn, leads to diagnostic delay and unneeded exposure to immunotherapy. Aside from biochemical testing, muscle biopsy, and genetic testing, the exercise intolerance and rhabdomyolysis occurring in mild forms of dysferlinopathy may be differentiated from metabolic myopathies by the persistently elevated CK, as compared to the fluctuating levels and intermittent normalization in most metabolic myopathies [11]. Given the rarity of these dysferlinopathy presentations, increasing awareness among clinicians is essential to facilitate early recognition and avoid misdiagnosis and unnecessary tests.

## Conclusions

Dysferlinopathy should be considered in the differential diagnosis of asymptomatic hyperCKemia and metabolic myopathies. The long-standing history of hyperCKemia without development of weakness over more than two decades in patient 1 suggests that asymptomatic or minimally symptomatic hyperCKemia in dysferlinopathy does not necessarily predict progression to muscle weakness. The lack of dystrophic features on muscle biopsy does not exclude a dysferlinopathy and should not halt the search for dysferlin deficiency in patients with asymptomatic hyperCKemia or exercise intolerance, especially in the setting of *DYSF* variants. Additionally, in the presence of such mild phenotypes, should a *DYSF* pathogenic variant be detected, it would be prudent to search for dysferlin deficiency in muscle, as a second *DYSF* variant could be missed by NGS (e.g., variant in non-coding regions).

## Data Availability

All patient data has been anonymized, and any further information may be obtained from the corresponding author.
